# A Case Report of Vanishing Bile Duct Syndrome Resulting From Thyroid Storm–Related Liver Injury and Postsurgical Biliary Tree Injury Overlap

**DOI:** 10.1155/crhe/7408410

**Published:** 2025-10-23

**Authors:** Joel Gabin Konlack Mekontso, Akil Olliverrie, Jingwei Ren, Nikolas St. Cyr, Vera Platsky, Joshua Diaz, Sara Samad, Shahbaz Khan, Michael Bernstein

**Affiliations:** ^1^NYC Health + Hospitals South Brooklyn Health, Brooklyn, New York, USA; ^2^Touro University College of Osteopathic Medicine, Harlem, New York, USA

**Keywords:** Graves' disease, postsurgical biliary tree injury, thyroid storm–related liver injury, vanishing bile duct syndrome

## Abstract

This case involves a 46-year-old obese, prediabetic female with a complex medical history. It illustrates several diagnostic challenges, including differentiating between thyroid storm–related liver injury and underlying chronic liver disease. The gradual progression to a definitive diagnosis of vanishing bile duct syndrome required multiple interventions and extensive evaluations.

## 1. Introduction

Vanishing bile duct syndrome (VBDS) is a rare, acquired, and potentially severe form of chronic cholestatic liver disease characterized by the progressive destruction and loss of interlobular bile ducts, referred to as ductopenia [1]. VBDS often presents as a complication of acute drug-induced liver injury (DILI), or other hepatobiliary insults, manifesting months after the initial hepatic insult.

While VBDS accounts for a small fraction of small biliary duct diseases, it is crucial to recognize its diverse potential causes and the severe implications it can have on patient outcomes. Early identification and intervention are essential for improving the prognosis and quality of life. In this report, we present a complex case of VBDS in a 46-year-old female with a history of postsurgical biliary tree injury and thyroid storm–related liver injury.

## 2. Case Presentation

This case involves a 46-year-old Hispanic obese, prediabetic female with a medical history that includes a cholecystectomy performed in Honduras in 2010, complicated by a bile duct injury that required a Roux-en-Y hepaticojejunostomy. In 2012, she underwent endoscopic retrograde cholangiopancreatography for postsurgical biliary stricture dilation.

In March 2020, she began receiving treatment in the United States, presenting with right upper quadrant (RUQ) abdominal pain, nausea, vomiting, and chills, but no fever. Laboratory results revealed a cholestatic pattern of liver damage (see Column 2 in Table 1), with normal lipase and total bilirubin.

**Figure 1:**
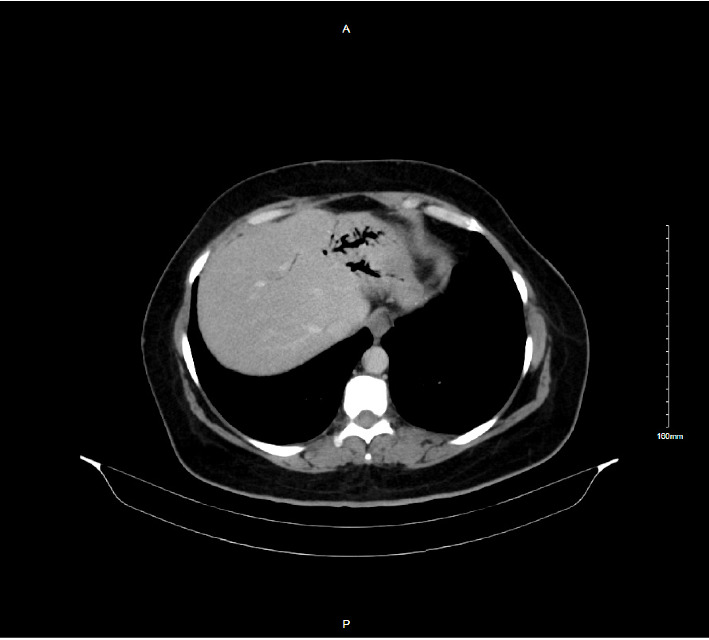
Abdominal CT scan (transverse section) demonstrating air within the left hepatic duct (pneumobilia).

An abdominal CT scan showed a normal-sized common bile duct, air in the left hepatic duct, and minimal peripancreatic fluid collection without stones (Figure 1). She was successfully managed as biliary pancreatitis with intravenous fluids, NPO, antibiotics, and ursodeoxycholic acid (UDCA) 250 mg twice daily to prevent gallstones. The viral hepatitis panel was negative.

In June 2020, she was admitted for thyroid storm with deranged liver function tests (see Column 3 in Table 1). She was diagnosed with Graves' disease and initially managed with propylthiouracil (PTU) at 200 mg every 8 h, chosen for its rapid onset of action. After a week, she was discharged on methimazole 15 mg daily, later increased to 20 mg daily for maintenance due to a better adverse effect profile. Follow-up 1 month later showed transaminase normalization (see Column 4 in Table 1).

In October 2020, she was readmitted for recurrent thyroid storm due to medication noncompliance, presenting with severe derangement in liver biochemistry (see Column 5 in Table 1), potentially related to the thyroid storm or DILI. On this admission, she received a single dose of 500 mg of PTU before being transitioned to methimazole by the endocrinology team. After the resolution of the episode, she underwent thyroidectomy and was initiated on levothyroxine. Thyroidectomy was performed to stabilize thyroid function and mitigate the risk of further drug or hyperthyroidism-induced liver damage.

One month after thyroidectomy, transaminases normalized, but ALP levels remained elevated (see Column 6 in Table 1).

**Figure 2:**
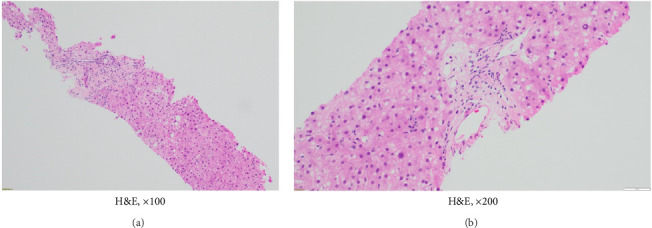
Liver biopsy showing a portal tract with loss of bile duct, hematoxylin-eosin stain, magnification × 100 (a) and × 200 (b).

**Figure 3:**
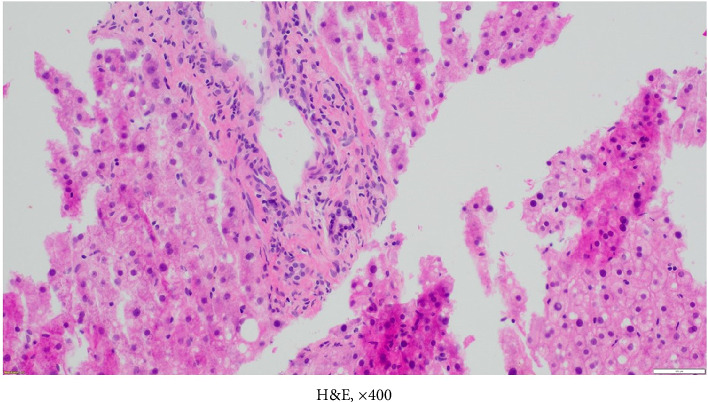
Liver biopsy showing a portal tract with bile duct injury, hematoxylin-eosin stain, magnification × 400.

**Figure 4:**
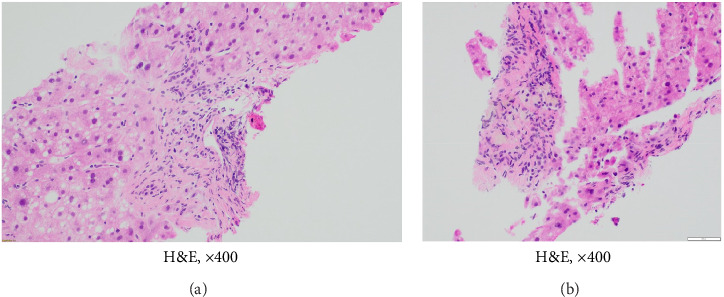
Liver biopsy showing a portal tract with bile duct injury (a) and loss of bile (b), hematoxylin-eosin stain, magnification × 400.

**Figure 5:**
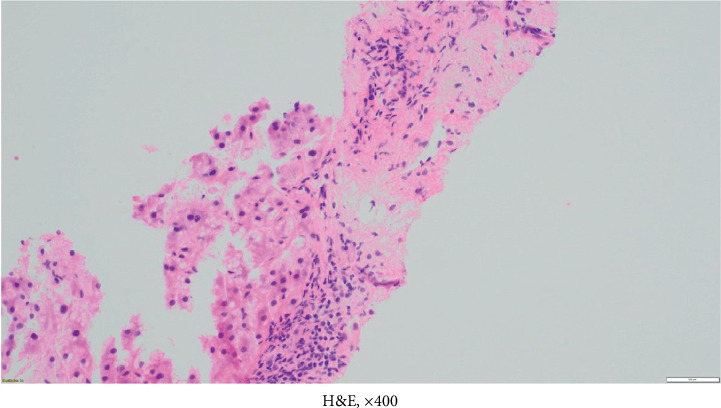
Liver biopsy showing a portal tract with bile duct injury and ballooning degeneration of hepatocytes, hematoxylin-eosin stain, magnification × 400.

**Figure 6:**
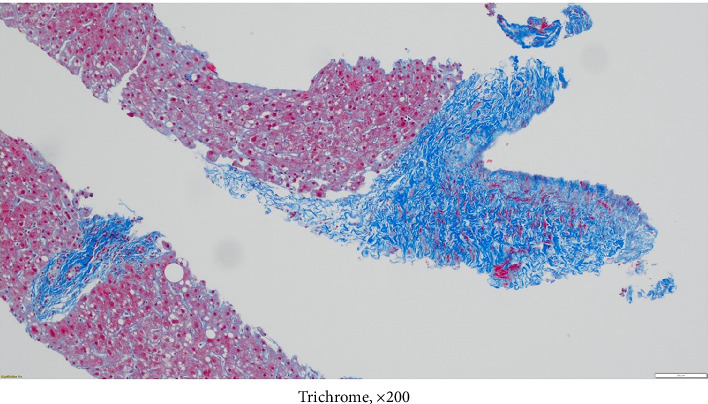
Liver biopsy showing portal and periportal fibrosis, trichrome stain, magnification × 200.

Throughout 2021 and 2022, extensive evaluations by the hepatology team excluded infectious, granulomatous, genetic, or autoimmune etiologies. Persistent cholestasis (see Column 7 in Table 1) led to a liver biopsy in July 2023, revealing severe ductopenia (< 50% of interlobular bile ducts in portal tracts), consistent with VBDS (Figures 2, 3, 4, 5, and 6). UDCA was restarted; the patient was offered the hepatitis B vaccine and advised to abstain from alcohol. Lifestyle modifications were also emphasized.

At her last follow-up in February 2024, she was asymptomatic with mild ALP elevation (see Column 8 in Table 1). Over the years, this patient has faced numerous life-threatening challenges and undergone extensive diagnostic and therapeutic interventions. These experiences have profoundly impacted her life and overall quality of life. However, with a definitive diagnosis now established and appropriate treatment initiated, she feels a sense of fulfillment and remains hopeful for the future, currently being symptom-free.

## 3. Discussion

VBDS refers to a broad group of disorders leading to the destruction of intrahepatic bile ducts [1]. In our case, it arose from a combination of postsurgical biliary injuries and thyroid storm–related liver injury. The repeated insults to the biliary system from multiple factors led to progressive damage and the subsequent loss of intrahepatic bile ducts, a condition known as ductopenia [2, 3].

VBDS is a rare condition, accounting for 0.5% of small biliary duct diseases [4]. A 10-year cohort study of 363 patients, published by the DILI network in 2017, focusing on liver injuries caused by drugs, herbal products, and dietary supplements, identified 26 patients with bile duct loss on liver biopsy, of whom 14 (3.8%) displayed moderate to severe ductopenia [5]. However, this study did not account for other potential VBDS etiologies. The majority of these patients (77%) were White, and 23% were Black, highlighting a racial distribution but not establishing a clear link to ethnicity [5]. In terms of gender, VBDS shows a slight female predominance, with some studies reporting a female-to-male ratio as high as 3:1. Notably, in both this cohort and our case, the diagnosis occurred in the fifth decade of life [5–7]. These findings highlight the variability in demographic patterns across studies and suggest that factors such as genetics or healthcare access may influence disease onset and progression.

The role of genetics in VBDS remains unclear. Research has demonstrated that IκappaB kinase (IKK) Proteins 1 and 2 are crucial for maintaining the bile–blood barrier and protecting the liver from cytokine toxicity and bile duct disease. Their combined deficiency leads to a disease resembling VBDS in mice, suggesting that similar genetic or molecular disruptions in humans could contribute to the development of this condition [8].

The pathogenesis of VBDS is poorly understood but has been linked to a variety of causes, including drug reactions, congenital, genetic, and immunologic disorders, infections, neoplasia, ischemia, toxins, and allograft rejection. In some cases, no clear cause is identified, and the condition is referred to as idiopathic [2]. To our knowledge, this is the first reported case of VBDS secondary to postsurgical injury of the biliary tree. This association merits further attention, particularly as biliary complications occur in approximately one-third of cholecystectomies [9].

Several mechanisms have been proposed for VBDS, including mitochondrial dysfunction, neoantigen formation, and cytokine-mediated apoptosis. A T-cell-mediated immune response may lead to antigen recognition on the biliary epithelium, resulting in immune cell infiltration, apoptosis, and T-cell cytotoxicity [2–4]. PTU, due to its hepatic metabolism and biliary excretion, was initially considered a potential contributor, an idea supported by the patient's Graves' disease (an autoimmune condition) and reports of positive lymphocyte stimulation tests for PTU in cases of DILI [10–12]. However, the patient's pre-existing liver abnormalities precluded the use of the RUCAM score [13]. This, combined with the temporal relationship between the patient's thyroid storm and liver injury, pointed toward an alternative etiology. We propose that the direct postsurgical biliary injury, exacerbated by the proinflammatory environment of the thyroid storm, played a significant role in the progression to VBDS.

VBDS may have a silent course in some patients; however, it may also present with symptoms of cholestasis such as jaundice, pruritus, and fatigue, often appearing 1–6 months after liver injury. Patients may also experience hypercholesterolemia and skin xanthomata. Laboratory findings commonly include persistent elevation of ALP despite normalization of serum aminotransferases. VBDS is suspected when ALP levels remain elevated for more than six months following liver injury, with no evidence of primary biliary cholangitis, sclerosing cholangitis, or graft-vs-host disease. A liver biopsy performed at least one month after injury showing less than 50% bile ducts in at least 10 portal areas confirms the diagnosis [14].

The primary approach to managing VBDS is to discontinue the offending agent, if identified, to allow for potential spontaneous recovery and to prevent further liver damage. Preventive measures include advising patients to abstain from alcohol, vaccinating them against hepatitis A and B, and ensuring proper nutrition with supplementation of essential vitamins (A, D, E, and K) and calcium [14]. Symptomatic treatment, such as antihistamines for pruritus, can improve sleep disturbances. Bile acid resins may be effective but are limited by gastrointestinal side effects. While the role of corticosteroids remains unclear, UDCA is commonly used for its presumed antioxidant and cytoprotective properties. However, evidence suggests limited impact on liver-related morbidity or mortality [5]. Plasmapheresis and calcineurin inhibitors have been used in some studies with variable outcomes [2, 4, 6].

The clinical course of VBDS is variable, ranging from resolution to complete loss of bile ducts and liver failure, potentially culminating in liver transplantation or death. A Chinese cohort study of 183 patients followed for a median of 4.8 years revealed that 88 (48%) progressed to cirrhosis, 27 (15%) died, and 15 (8%) underwent liver transplantation. Notably, 42 (23%) experienced poor outcomes within two years of diagnosis [7].

Several factors correlate with a more severe disease course. Although not statistically significant, younger age and Black race have been associated with poorer outcomes in some studies [5]. In contrast, other studies have linked older age to worse outcomes [6]. Decreased liver synthetic function, elevated bilirubin (median 18.5 mg/dL), and ALP (median 746 U/L) at biopsy portend poorer outcomes (*p*=0.04) [5, 6]. Histopathological findings such as hepatocellular cholestasis, foam cells, advanced fibrosis, and extensive bile duct loss correlate with more severe disease [5, 7]. Our patient, who did not present with these risk factors, will continue to be monitored beyond the critical 2-year period.

The management of VBDS often requires a multidisciplinary approach. In our case, this involved internal medicine, hepatology, endocrinology, and surgery. Early recognition of the condition and prompt identification and mitigation of potential contributing factors are crucial for improving outcomes. Clinicians should remain vigilant for signs of cholestasis, particularly in patients with a history of postsurgical biliary injury. Regular monitoring of liver function, especially ALP, and timely liver biopsy are a key to confirming the diagnosis. When determining the optimal management strategy, clinicians must conduct a thorough evaluation of all possible causes of persistent cholestasis, including drug-related liver injury, genetic predisposition, autoimmune conditions, and prior surgical interventions.

## 4. Conclusion

The patient's clinical course—marked by multiple life-threatening events, including thyroid storm, evolving hepatic dysfunction, and VBDS—highlights the necessity of early recognition, vigilant monitoring, and timely intervention. Additionally, this case reinforces the value of thorough diagnostic evaluation to differentiate potential etiologies of liver injury and optimize management strategies. Beyond medical treatment, patient education and supportive care play a crucial role in improving outcomes and enhancing long-term quality of life.

## Figures and Tables

**Table 1:** Changes in liver chemistry over time.

Laboratory tests	Mar 2020	Jun 2020	Jul 2020	Oct 2020	Nov 2020	Jul 2023	Feb 2024	Normal range
Albumin (g/dL)	4.4	4.1	4.0	4.4	4.6	4.4	4.4	3.5–5.0
Aspartate aminotransferase (U/L)	298	170	34	330	21	30	22	≤ 35
Alanine aminotransferase (U/L)	139	75	28	232	21	48	22	≤ 35
Alkaline phosphatase (U/L)	249	240	253	513	274	176	160	35–104
Gamma-glutamyl transferase (U/L)	—	—	—	800	—	—	—	6–42
Total bilirubin (mg/dL)	1.2	1.1	0.3	1.3	0.4	0.3	0.4	0.2–1.2

## Data Availability

The data that support the findings of this study are available on request from the corresponding author. The data are not publicly available due to privacy or ethical restrictions.
